# Social Connectedness in Family Social Support Networks: Strengthening Systems of Care for Children with Special Health Care Needs

**DOI:** 10.5334/egems.232

**Published:** 2018-11-23

**Authors:** Danielle M. Varda, Ayelet Talmi

**Affiliations:** 1 Visible Network Labs and University of Colorado, US; 2 University of Colorado School of Medicine, US

**Keywords:** electronic health records, social networks, organizational innovation, quality improvement, data collection

## Abstract

Current approaches to addressing the problems families face when navigating complex service systems on behalf of their children rely largely on state or nationally driven efforts around the development of systems of care (SOCs). However, operationalizing meaningful family involvement within SOCs remains a challenge, with little attention paid to the role of personal social support networks (PSSNs). Specifically, risk factors related to the variations in the social connectedness of family social support networks are difficult to identify, assess, and track over time. This paper summarizes families’ descriptions of their PSSNs and describes the development of a social network analysis tool, the Person-Centered Network App (PCNA), used to measure and monitor the social connectedness of families of children with special health care and developmental needs. Twenty-nine families participated in the project and completed social network surveys, identifying a total of 38 unique types of support partners and 230 partnerships (dyadic relationships). Families identified a range of formal and informal members including primary care providers, medical specialists, family, friends, faith-based organizations, insurance providers, nurses, community organizations, early interventionists, school resources, other families, online support groups, and public resources, rating 61 percent of them as “very important.” Informal network members (e.g., family, friends) provided emotional and day-to-day support. Primary care providers, medical specialists, and public resources provided health care services while early intervention and medical specialists provided therapies. PSSNs were characterized by high levels of trust but low levels of coordination. These findings inform providers and case workers that families can readily describe their social connectedness in ways that may affect health care access and utilization. Understanding how PSSNs function in the lives of families of children with complex health care needs provides opportunities for improving systems of care (e.g., medical homes) and ultimately, enhancing health and developmental outcomes.

## Introduction

In the United States, more than 12 million children are identified as having special health care needs [1]. In 2011–2012, 19.8 percent of U.S. children under the age of 18 had a special health care need, representing 14.6 million children [2]. Previous research indicates that among households with children under the age of 18 years, nearly one quarter (23.0 percent) include at least one child with special health care needs [3].

Current approaches to addressing the problems families face when navigating complex service systems on behalf of their children rely largely on efforts to fund state or nationally driven systems of care (SOCs). Such efforts, often implemented at the local level, are often designed as network interventions, coordinating services and creating an integrated, connected system spanning independent sectors [4]. Since Stroul and Friedman’s [5] original monograph defining SOCs, considerable investment has been made in creating systems of care to effectively address the complex needs of children with serious mental health challenges. In 2010, Stroul, Blau, and Friedman [6] updated the definition of a SOC, describing it as “a spectrum of effective, community-based services and supports for children and youth with or at risk for mental health or other challenges and their families, that is organized into a *coordinated network, builds meaningful partnerships with families and youth*, and addresses their cultural and linguistic needs, in order to help them function better at home, in school, in the community, and throughout life,” (p. 6). Demand is growing for interventions that also address the social determinants of health and bridge social and medical care in pediatric clinical settings, including screening for and addressing unmet social, legal, and mental/behavioral health needs of families seen for primary or urgent care [7].

Inherent to the SOCs approach are the guiding principles of social connectedness that include individualized, community-based, accessible, family-driven and youth-guided, coordinated services that consider family, friends, and cultural factors [8]. Among these resources are both an individual’s informal social supports *and* the formal social service systems such as those of child welfare, education, corrections, and health care [9]. Yet specific attention to the role that families’ personal, largely informal social support networks play in SOCs has been neglected in research and, often, in practice. In fact, operationalizing meaningful family and friend involvement within SOCs remains a challenge, leaving many communities and providers struggling to implement patient-driven frameworks [10]. Although evidence exists to show that SOC approaches [11] hold significant promise in addressing the needs of children who struggle with serious mental/behavioral health challenges (e.g., improved coordination and responsiveness to client needs through provision of wraparound services), such systems-level efforts may be far removed from the manner in which personal social support networks created by families operate.

While SOCs efforts are growing, they lack three important elements that can inform their effectiveness and ability to integrate and leverage the informal social connections of families:

1. broad characterizations of systems that include proximal and distal influences through formal and informal networks;
2. an analytic approach to evaluate systems of care, which are inherently dynamic and highly individualized at both the community and personal levels; and, subsequently,
3. an empirically based tool for providers to assess personal support networks and refer families to system resources when gaps are identified, to enhance individual system functioning and, ultimately, to achieve better population and individual outcomes.

In fact, one of the greatest challenges in integrating a family’s informal personal support networks into their SOCs lies in the difficulty in identifying, assessing, and tracking these informal networks over time. No standard assessment or methodology has been integrated into formal care and treatment for families, one that can create an integrated “whole network of care” plan for that family, leveraging both their formal and informal resources to achieve greater health and mental/behavioral health outcomes.

In this paper, we describe how SOCs can be characterized using a method known as social network analysis and how this type of information can be used within clinical and community-based settings to assess the strengths and gaps in a family’s social support network and integrate those resources into the formal system of care to create a care plan for families that can impact health and mental/behavioral health outcomes. We answer two primary research questions:

- “How can we better understand the informal social support networks of families of children with special health care and developmental needs?”
- “How can we systematically collect data to reliably assess variances in social support, and, in turn, build adaptable systems to address the many diverse needs of families?”

With data to answer these questions, providers and community organizations can use tools that integrate social network data, collected from patient and their families, to engage in care plans, decision-making, and creation of interventions that can identify and be used with families who have a higher risk of adverse social connectedness. This method and type of assessment is meant to help a provider distinguish those who are at greater risk of adverse social connectedness, and those who have more strength from strong personal connections. Therefore, those with greater risk (those, for example, who are estranged from their families or have few friends) will be more “visible” to the provider, with this approach.

## Methods

To explore how families, describe their personal social support system networks, we collected social network data from families to characterize the way they articulate their social connectedness related to navigating complex systems of care. Social network analysis (SNA) is a novel way to collect information to better understand families’ social support systems as they interact in formal SOCs. SNA is a quantitative methodology that focuses on relationships between and among social entities, measuring and mapping relationships and flows between people, groups, or organizations [12]. This approach can identify the members of a person’s social support network, the quantity and quality of relationships among those members, and other characteristics such as types of interactions, resource exchange, reciprocity, and trust among members [13,14]. Collecting network data allows for visualization of the connections among stakeholders and clients using network maps. Unlike standard social and behavioral science statistics, variables collected in SNA are functions of relationships between entities. As such, data arising from network studies apply to the analysis of relational data measured on dyads or groups of social actors. In this paper, data are collected and analyzed to describe:

- the composition of personal social support networks (e.g., both formal – service providers, funders, community programs, state departments, and informal – family, friends, neighbors, churches, etc.),
- the number and quality (trust and value) of relationships among stakeholders,
- the contributions made by each stakeholder (e.g., services, technical assistance, information exchange), and
- the levels of coordination among those stakeholders.
- The study was designed in three phases as an in-depth exploratory approach to test a data collection tool for characterizing personal social support networks and measuring social connectedness. Families were recruited for all phases to participate in design of the measures and in administration of the tool in two interactions.

Additionally, the WONDERbabies (Ways of Nurturing Development through Enhancing Relationships) Partnership for Health Collaborative (a statewide stakeholder network in Colorado that includes organizations, programs, providers, and services—all working together to enhance systems of care for babies and young children with special health care and developmental needs) actively participated in all stages of this project, providing data for the SNA and implementing systems change based on the results. The WONDERbabies partners were active in recruiting families to participate in two case site studies (described below) for data collection to assess individual families’ personal social support networks and their interactions within formal SOCs.

This project and the activities detailed below were approved by the Colorado Multiple Institutional Review Board. Participant consent was obtained at the time of data collection. Families received a $20 gift card for their participation.

### Developing the Instrument to Assess Families’ Social Support Systems

This study administered and pilot tested several versions of a survey to assess a family’s personal social support system. In phase 1, a focus group was held to hear how families articulate and conceptualize their social support networks. In phase 2, a questionnaire was administered that included a limited set of questions that were given to families, with room for open-ended responses. The results from phase 2 informed the redesign of the questionnaire for phase 3. The questionnaire administered in phase 3 asked families to describe their children, relationships to others who help them care for their children, and to rate those others on levels of trust, support, importance, and coordination. The results of this study led to a third and final redesign of the questionnaire that is now referred to as the Family Social Support Survey, part of the Person-Centered Network App’s (PCN App’s) suite of social connectedness assessments. The PCN App is a social network data collection and analysis tool, part of the ASPEN platform (a network intervention platform), designed for the purpose of collecting social network data at the ego-centric (personal network) level.

### Collecting Data in Three Family-Centric Sites

Three sites were chosen to recruit families to pilot the PCN App. The three site studies were conducted subsequently to give the research team time to engage patients as stakeholders in the research process, give feedback on the process and data results, and inform subsequent administration of the Family Social Support Survey. The survey was designed, modified, and expanded following each site visit, based on analysis of the data and feedback after analysis was complete.

**Site 1 (Phase 1):** As a first step in the study, we recruited a group of parents who have children with special health care and development needs to participate in a focus group where we asked them to describe their “ideal” system of care and to draw their personal social support networks that help them coordinate care for their children. From this focus group, we developed a list of areas to include in our first questionnaire, and developed our initial assumptions that parents rely primarily on their informal networks of care as a preference to utilizing formal system resources.

**Site 2 (Phase 2):** Following the focus group at the first site, we recruited a purposeful sample of 10 families chosen from a list of participants nominated by WONDERbabies Partners and invited to participate in data collection efforts. Families were asked to answer the initial questionnaire and follow up questions were utilized to allow the research team in-depth understanding and interpretation of results. These 10 families provided data on 88 relationships.

**Site 3 (Phase 3):** At the third site, 21 families participated in the study at a family clinic serving Spanish- and English-speaking families with children with special health care needs and asked to participate. The survey was translated into Spanish for families that were not English speaking. A navigator was present during each family’s responding to answer any questions. These 21 families provided data on 125 relationships.

The final number of relationships between families and both formal/informal support network relationships include an n = 208 relationships, providing a rich sample of the quality and quantity of informal and formal social support networks of families of CSHDN. The results below are based on analysis these 208 relationships, with the goal to demonstrate a systematic methodology to collect data and characterize social support networks of families, as a tool to inform provider knowledge and care giving.

## Results

### Parent Focus Group (Study Site 1)

**Figure 1:**
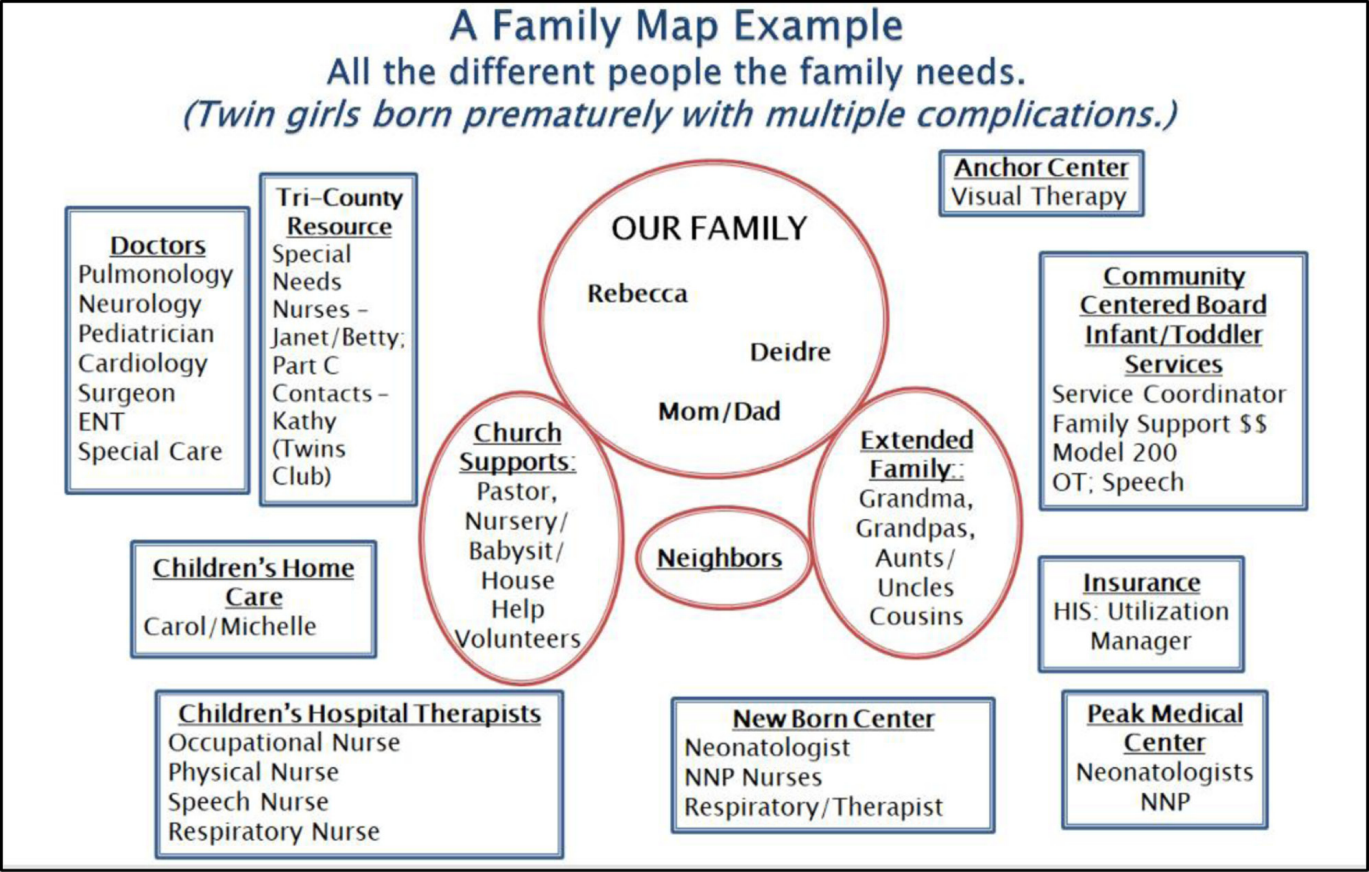
Personal network of care created by a family with twins with special health care needs.

Findings from the focus group included: parents strongly asserting that they prefer to rely on informal supports whenever possible and that more formal resources were necessary as needs became more complex. Families relied on each other and had varying ways to describe their personal social support networks. Parents also expressed a desire to have early education on the needs of special needs children integrated into their pre-natal care experiences. Families were asked to draw their systems of care. An example of a drawing a parent might have produced is below in Figure 1. This illustration was typical of other families, where SOC are complex but that the “closest” connections are primarily informal.

### Family Social Support Networks (Study Site 2)

All 10 family respondents were mothers of children ranging in age from 15 months to 17 years who lived in an urban area of the state. Most children were diagnosed with a developmental delay, two had medical conditions, two experienced social/emotional delays, and almost all required additional or special care giving daily. These 10 families identified a total of 38 unique types of support partners and a total of 88 partnerships (dyadic relationships) as part of their family support networks.

Family support networks were comprised predominantly of informal, personal connections such as family, friends, online support groups, schools, and pediatricians (a formal/professional relationship). We found surprising variations in responses to value and trust questions within family support networks. While family members, friends, primary care providers, and school-based resources were mentioned most often as “very important” to the family (89 percent), only family members were mentioned as playing an important role in working with other members of the network a “great deal” (67 percent), while over half of the respondents noted that primary care providers worked with others “not at all” (33 percent) or “a small amount” (22 percent). Families varied in terms of perceptions of trust. All family members were trusted (100 percent) and were also identified as providing “a great deal” of trust (22 percent). Medical specialists (17 percent) and PCPs (15 percent) also provided “a great deal” of trust. Importantly, the highly valued and trusted organizations at the state systems-level did not appear as frequently, if at all, in family support networks with the exception of primary care providers.

No pattern was found in the way families characterized their personal social support networks, even when compared across child’s diagnosis, location of services received, or time in the system. The only pattern determined was a common theme of listing primarily informal supports as the most important members of their system of care, and a preference for self-sufficiency throughout the process of caring for their children. Pediatricians or specialists were listed as the second most important type of member of families’ systems of care. At the completion of the Site 2 data collection and analysis, the survey questions were revised and expanded to incorporate findings and feedback from families and stakeholders for pilot administration in Site 3.

### Family Social Support Networks (Study Site 3)

**Respondent Information.** Twenty-two families participated in site study 3. Of those, 16 were the mother of the child(ren). Respondents reported that 82 percent had children with a medical condition, followed by 77 percent who reported a developmental delay (77 percent). Additionally, 77 percent said their children were technology dependent and 73 percent of their children used home health care. Fewer families reported that their children had limited mobility (27 percent), emotional or social delays (18 percent), or required a lot of health care needs/costs (14 percent).

The average number of relationships reported by families was 6, meaning that most respondents listed 6 other people when asked to “Please list all of the people, places, organizations, and other resources that you would include in your support network” (question 10 of the survey). Most relationships were with community organizations (24 percent), medical specialty providers (18 percent), family members (17 percent), and school-based resources (15 percent). Other types mentioned included primary care providers (8 percent), home visitors (7 percent), friends (6 percent), early intervention providers (6 percent), public resources (6 percent), insurance providers (3 percent) and others (2 percent).

**Figure 2:**
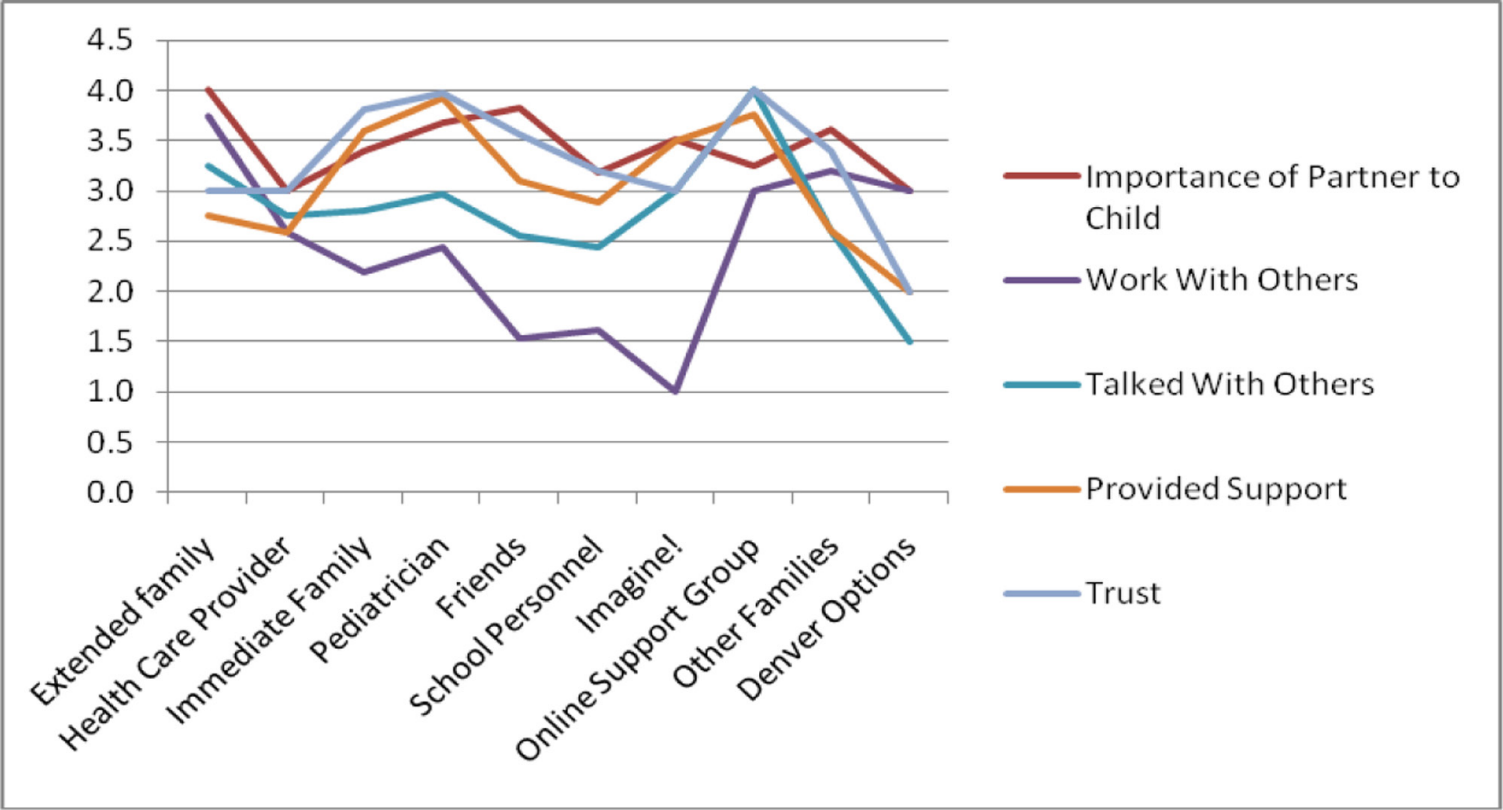
Combined Results of Families’ Descriptions of Social Support Networks, on a scale of 1–4 (1 = not at all, 2 = a small amount, 3 = a fair amount, 4 = a great deal).

**How do families describe the quality and nature of their personal support network relationships?** Types of support provided by social support networks included: emotional support (43 percent), therapy/intervention (25 percent), day-to-day support (23 percent), health care services (21 percent), other interventions (19 percent), financial support (14 percent), childcare (14 percent), other types of support (14 percent), and home-based services (8 percent). These results are reflected in Figure 2 below.

When asked to rank each relationship in terms of importance, trust, level of support provided, and coordination among people in the social support systems, results varied. Almost all relationships were rated as very important to the families and their children. Respondents also reported that members of their social support network provide a great deal of support (50 percent) or a fair amount of support (30 percent) for them and their children. Somewhat surprising, 19 percent of the relationships provided no or only a small amount of support. Trust seems to be fairly high in personal social support networks. In fact, 50 percent said they trusted their personal network members a great deal or a fair amount (33 percent). Again, surprisingly some were identified as only trusted a small amount (10 percent) or not at all (6 percent).

While relationships were described as important, supportive and trusting, respondents did report that people in their social support systems are unlikely to talk together or work together to coordinate care for their children. Over half of the relationships were described as only knowing or working together a fair or small amount (59 percent). Sixteen percent said others in the network do not know each other at all. When asked if members of a family’s personal network talks to others in the network, 68 percent reported that this only happens a fair amount or less. Only about 31 percent said this occurs a great deal.

**Perception of Success.** When asked how successful the families felt the system of care is at providing coordinated, comprehensive care for your child, 55 percent said that the SOC is only somewhat or not successful. Twenty-three percent of those reported that the SOC is not successful. Forty one percent said they felt the SOC was successful or very successful.

**Figure 3:**
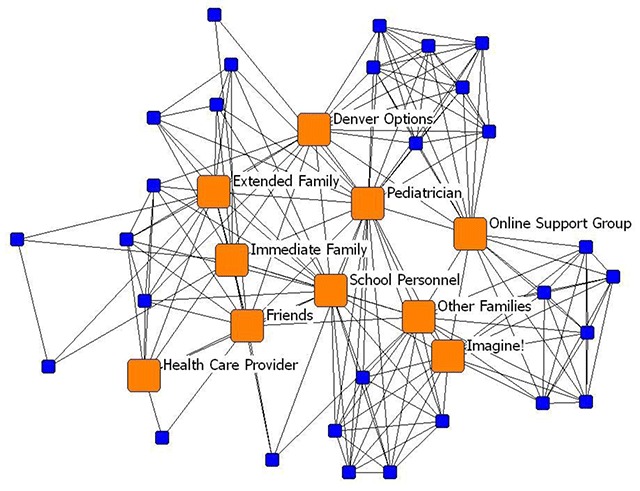
Family Social Support Networks Nodes as support and lines as families who share those types of supports in their networks.

**Personal Networks Mapped Out.** Family support networks are depicted as individual respondents’ “ego-networks” (personal network) and this type of data collection allows us to create “network maps” of the data. Ego-networks only show the relationships between the respondent and the network members that they nominated as providing personal support for their families. However, in the map below (Figure 3), we combined all of the respondents’ ego-networks into one map and transformed the data so that the nodes represent that members of their personal networks, and the lines represent where respondents most often mentioned common types of members of their social support network (represented by the lines (to indicate their shared relationship types)). Labels were given to those members of the network that were picked by three or more families. This processed allows us to look for commonalities and trends across networks. In this map, several “key players” (orange square nodes) were identified as appearing commonly in social support networks.

## Discussion and Recommendations

Overall, the findings reported in this paper provide a range of information on how families describe their personal support networks and allow us to answer our research questions: *“How can we better understand the informal social support networks of families of children with special health care and developmental needs?”* and, *“How can we systematically collect data to reliably assess variances in social support, and in turn, build adaptable systems to address the many diverse needs of families?”*

### “How can we better understand the informal social support networks of families of children with special health care and developmental needs?”

While the data show expected results on the characteristics of the caregivers and the types of special health care and developmental needs families are navigating, the ways in which families describe their social support networks had some surprising results, specifically around the lack of coordination families experience, even among their close personal connections. The main learnings from this work show us that:

- **Families Rely Primarily on their Informal Supports:** Families have a preferential tendency for leveraging their informal supports *over* accessing systems resources (indicated by the few mentions of formal supports in their personal social support networks), suggesting that they are more inclined to develop ways to help themselves through informal mechanisms before accesing formal systems resources. This finding is particularly poignant in the increasing efforts of providers and community organziations working hard to build systems of sare for families. If families are relying on their informal supports, and these are not incorporated into their SOCs, we are unlikley to create integrated “whole network of care” plans that leverage all of the available assets a family might have to be successful.
- **Famlies Report Trusted and Important—Yet Unccordinated—Supports.** While families tend to report that a combination of their formal and informal supports are important and trusted members of their social support systems, creating a web of social supports, they also reported that most of these social support members are not connected to one another, do not talk to one another, and even fall a little on reports of providing support. This is an important finding because reseach has demonstrated that coordinated systems of care have a positive impact on health and mental/behavioral health outcomes. While familes are able to identify trusted, important, and supportive connections, there is a clear and strong pattern that these connections are not working together in a coordinated way.

While this was a trend across the families and these relationships, it was also clear that it will be difficult to categorize Family Social Support Networks into simple buckets to incorproate into clinical treatment and social service referrals. Rather, individualized assessments can give providers a way to personalize care and referrals to systems resources, enhancing patient-centered solutions. With increasing access to systematic, reliable measures of social connectedness, it will be more and more possible for providers to create customized care plans and adapatble solutions (interventions) for their patients. As we move this type of assessment (and research) forward, we can continue to build the data on these types of social support systems, and begin to build the evidence-base to prioritize referrals that are both indivdualized and cost-saving to the system.

### “How can we systematically collect data to reliably assess variances in social support, and in turn, build adaptable systems to address the many diverse needs of families?”

**Figure 4:**
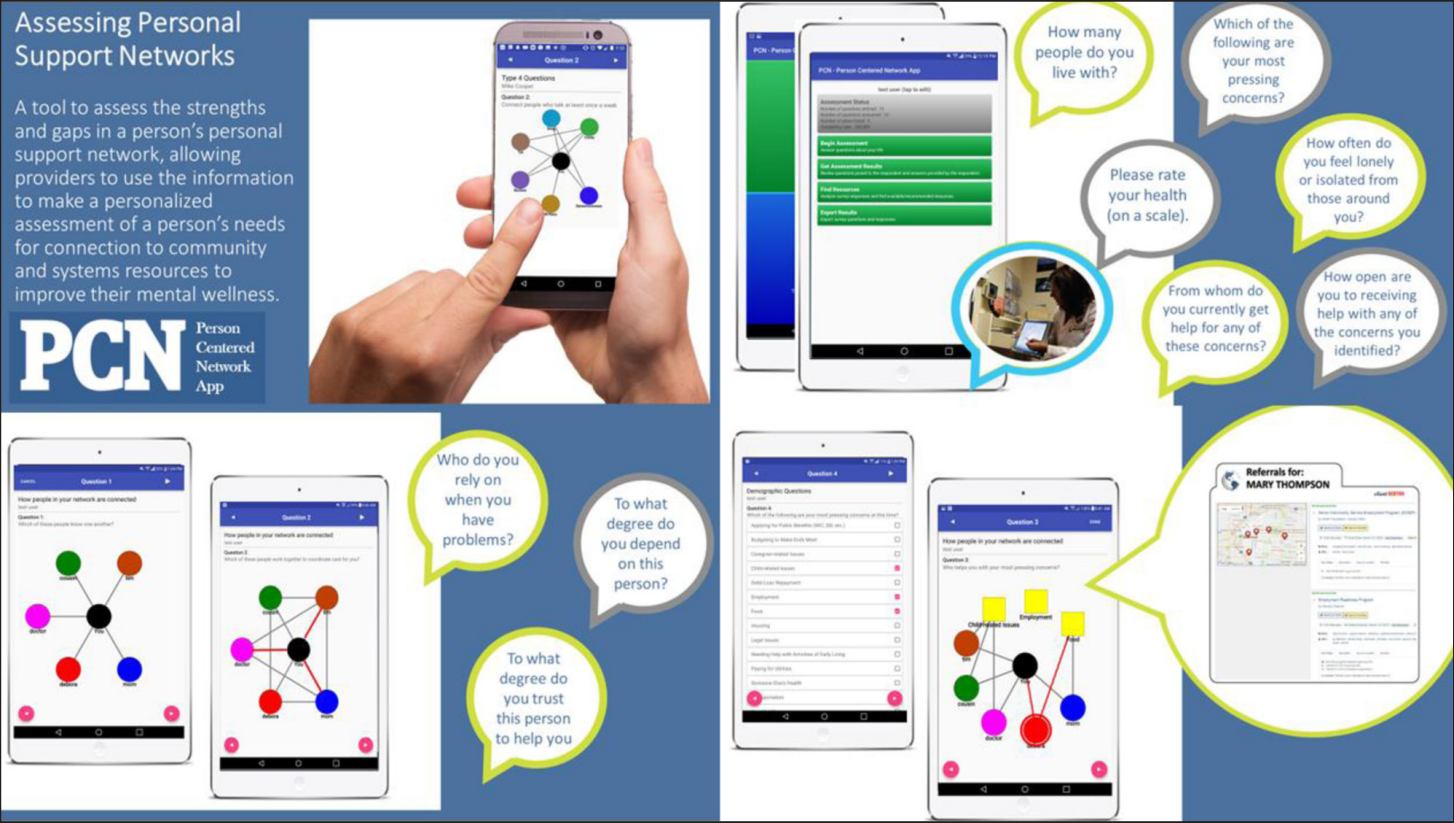
Screenshots of the Person-Centered Network App. See https://www.visiblenetworklabs.com/pcn-app/.

After several interactions of survey pilot testing, the Family Social Support Survey is now programmed in the Person-Centered Network App (PCNA) on the ASPEN platform. The PCNA is an interactive assessment tool for use by a provider (for example, a doctor, a social worker, a nurse, or even front-line staff) to first screen a person to assess their gaps and strengths in their personal support systems and then, based on the results, link them to available community resources (See Figure 4). The PCN App allows a provider to work with a patient/client (a respondent) to collect data on a person’s personal social support network, identifying who they are connected to and how well connected (or not) those network members are to one another. It also assesses the content, strength, and importance of those connections. The respondent uses a touch screen to answer questions and draw a visual of their personal social support network, making the tool patient-centered by empowering the patient to be involved in the assessment and diagnosis of variations in social connectedness.

The current version of the Family Social Support Survey (currently being beta tested by a larger population of families) asks respondents to rate their own health, feelings of loneliness, their most pressing need (social determinants of health), readiness to receive resources, and identify social relationships that are part of their personal social support networks. They are then asked to rate those relationships on levels of trust, support, importance, and level of coordination among them and then find the gaps where they have no help to connect them to systems resources. The assessment, which takes about 5 minutes to complete, produces a summary report of levels of social connectedness and recommendations and links to resources in real time (inside the app). The data collected with the PCN App are stored systematically on a HIPPA compliant server to track and store data over time, as well as track at the organizational and population health levels. Figure 4 shows screenshots of the PCN App. The goal of this tool is to develop a patient-centered empirically developed and validated way for providers to track the personal social support networks of their patients/clients to determine levels of risk of adverse social connectedness, and to priorities which interventions will be most effective for the variations in the ways families present across social and economic strata.

### What’s next for this research?

Families continue to be screened with the Family Social Support Survey at sites across the United States. A larger sample of data is being collected and validation of the tool is underway. A risk score related to variations in social connectedness is developed and will be tested as it relates to health, mental/behavioral health, and wellness outcomes.

Data provided by families about their personal social support networks can be combined with other types of health and mental/behavioral health data, providing a better understanding of how SOCs impact the daily lives of families and their ability to care for their children, coordinate services, and successfully follow through on health care instructions from providers. When families suffer from weak social connectedness, they are more likely to struggle with providing the care, comfort, and logistics necessary to address the complex social, emotional, and medical needs of their children. When providers have an in-depth understanding of the variations in social connectedness of their patients, provided through a systematic collection of information and a diagnostic tool to uncover new dimensions of SOCs, network interventions (such as systems of care approaches) can be designed to help fill gaps and leverage strengths for families. Providers currently have little way to know the quality, quantity, and content of the social support systems of the families they treat, making it challenging to know who is at higher risk of adverse social connectedness. Family Social Support Network data can be used to identify those families that need more systems support from other providers and community resources to assist families in coordinating referral to systems resources to fill gaps in personal support networks.

The next level of data analysis required will directly measures child and family outcomes at they relate to variations in social connectedness, a connection that is critical to evaluating the extent to which SOCs achieve such objectives. This requires gathering family support network data alongside data on child outcomes, and access to needed social, mental/behavioral, and medical resources among other variables to more directly link systems efforts (e.g., medical home initiatives) and family support resources with child and family outcomes. Further exploratory work includes developing definitions of “success” as perceived by both systems stakeholders and families who utilize the system.

Ultimately there is no perfect model of what a family’s “personal social support network” should look like, given the cultural, social, and variations in health diagnosis among patients. There is no “right” number of connections, although there is a combination of factors that provide diagnostics to mitigate high risk from lower risk. For example, being connected to a large number of others that are abusive, dependent, or addicted, would constitute high risk. Few connections to strong healthy connections constitute lower risk. This type of assessment can detect that difference. Therefore, assessing each person in an individualized way and linking their specific gaps in resources and support can improve preventive, promotional and early intervention approaches to mental wellness primarily in the context of early childhood. While early work to develop the PCN App evolved around children with special health care needs, this tool can increase our ability to both systematically assess the strength of a person’s personal support network, link the person to available community resources, and ensure more meaningful care of a patient’s mental wellness in almost any setting (e.g., chronic diseases such as cancer; specific populations such as aging, and other contexts such as homeless or adolescents).

### Limitations of Social Network Approaches

Several limitations exist when using social network analysis. One limitation involves the inability to correlate the *practice* of collaboration/coordination with, or to predict, population health outcomes. While the methodology introduced is novel and has potential to improve our ability to link systems of care efforts to outcomes, characterizing a family’s perception of how well their support network is coordinated as an “outcome” does not adequately measure population health or individual level health indicators as outcomes. However, ongoing work involves correlating county level health indicators with relationships among systems-level and family support networks to examine population well-being at the local level. Additionally, we are collecting individual health outcomes data (e.g., socio-demographic characteristics, diagnoses, utilization of health care services, referral uptake, etc.) and correlating network characteristics with these health outcomes. Data will be utilized in future efforts in targeted communities to enhance systems of care and effect change.

A second limitation includes the limited resources to continually increase the sample size (in a snowball sampling method) and the time-intensive requirement of network surveys. As with other data collection efforts, social network data are only as comprehensive as the respondents who choose to participate in characterizing the networks in question. Obtaining a 100 percent response from an unknown sample size is difficult. However, the use of an ego-centric (vs. whole network) design allows for sampling and a greater possibility to generalize.

## Conclusion

This work assumes that comprehensive and coordinated systems of care are vital to health outcomes including social-emotional development and family well-being. This assumption underlies SOCs funding and evaluation efforts over the past decade and beyond. Developing such systems depends upon strong collaborative efforts among a multitude of stakeholders. While funding and focus remains on formal, organizational stakeholders, largely ignoring the importance of informal personal networks (made up of family, friends, schools, pediatricians, and schools), we anticipate that without new evaluation tools that include examination at both the systems level and the client level, we will continue to see mixed results from families in terms of satisfaction and a continued lack of adequate support and coordination of systems of care designed to address the complex needs of children and their families. Social network analysis, when integrated into the work flow with tools such as the Person-Centered Network App, is a powerful, data-driven approach that can inform and enhance how systems of care function and ultimately, how systems of care impact the lives of the children and families they serve. To build the evidence base necessary to support this assumption, more research is needed to 1) characterize SOCs from the family perspective and 2) link these data to health outcomes. Integrating tools that will assess and systematically store these data as electronic health records on this topic (like the PCN App), utilized across a variety of health system and community based organizational settings can begin to address these issues and improve health outcomes for families.

## References

[1] Newacheck, PW, Strickland, B, Shonkoff, JP, Perrin, JM, McPherson, M, McManus, M, et al. An epidemiologic profile of children with special health care needs. Pediatrics. 1998; 102(1): 117–123. DOI: 10.1542/peds.102.1.117 9651423

[2] Child Health USA. Children With Special Health Care Needs; 2014 https://mchb.hrsa.gov/chusa14/population-characteristics/children-special-health-care-needs.html. Accessed October 29, 2017.

[3] Child and Adolescent Measurement Initiative. Data Resource Center. Accessed July 21, 2014.

[4] Hodges, S, Friedman, RM and Hernandez, M. Integrating the components into an effective system of care: A framework for putting the pieces together In: Stroul, BA and Blau, GM (Eds.), The system of care handbook: Transforming mental health services for children, youth, and families. 2008; Baltimore, MD: Brookes Publishing.

[5] Stroul, BA and Friedman, RM. A system of care for children and youth with severe emotional disturbances (Revised edition). Washington, D.C.: Georgetown University Child Development Center, CASSP Technical Assistance Center; 1986.

[6] Stroul, BA, Blau, GM and Friedman, RM. Updating the system of care concept and philosophy. Washington, D.C.: Georgetown University Center for Child and Human Development, National Technical Assistance Center for Children’s Mental Health; 2010.

[7] Gottlieb, LM, Hessler, D, Long, D, Laves, E, Burns, AR, Amaya, A, Sweeney, P, Schudel, C and Adler, NE. Effects of social needs screening and in-person service navigation on child health: A randomized clinical trial. Journal of the American Medical Association Pediatrics. 2016; 170(11): e162521 DOI: 10.1001/jamapediatrics.2016.2521 27599265

[8] Spencer, SA, Blau, GM and Mallery, CJ. Family-driven care in America: More than a good idea. Journal of the Canadian Academy of Child and Adolescent Psychiatry. 2010; 19(3): 176–181. DOI: 10.1111/j.1939-0025.2010.01067.x 20842272 PMC2938750

[9] Ungar, M. The social ecology of resilience. Addressing contextual and cultural ambiguity of a nascent construct. The American Journal of Orthopsychiatry. 2011; 81: 1–17. DOI: 10.1111/j.1939-0025.2010.01067.x 21219271

[10] Baxter, B. For families, actions speak louder than words. Evaluation and Program Planning. 2010; 33: 39–40. DOI: 10.1016/j.evalprogplan.2009.05.011 19541366

[11] Stroul, BA and Blau, GM. The system of care handbook: Transforming mental health services for children, youth, and families. 2008; Baltimore, MD: Brookes Publishing.

[12] Cross, R, Laseter, T, Parker, A and Velasquez, G. Using social network analysis to improve communities of practice. California Management Review. 2006; 49(1): 32–38 DOI: 10.2307/41166370

[13] Scott, J. Social Network Analysis: A Handbook. 1991; London: Sage Publications.

[14] Wasserman, S and Faust, K. Social Network Analysis: Methods and Applications. 1994; Cambridge: Cambridge University Press DOI: 10.1017/CBO9780511815478.

